#  Interplay Between the In-Vitro Cleaning Performance and Wear of Manual Toothbrushes in Fixed Orthodontic Appliances 

**DOI:** 10.3290/j.ohpd.b5883989

**Published:** 2024-12-18

**Authors:** Florance A. Lasance, Andrea Gubler, Thomas Attin, Florian J. Wegehaupt

**Affiliations:** a Florance A. Lasance Postgraduate Student, Clinic of Conservative and Preventive Dentistry, Center for Dental Medicine, University of Zürich, Plattenstrasse 11, CH-8032 Zürich, Switzerland. Research idea, experimental design, wrote the manuscript, performed the experiments in fulfilment of requirements for a doctoral degree.; b Andrea Gubler Head of Laboratory, Clinic of Conservative and Preventive Dentistry, Center for Dental Medicine, University of Zürich, Plattenstrasse 11, CH-8032 Zürich, Switzerland. Supported and organised the implementation of the experiments.; c Thomas Attin Professor and Director, Clinic of Conservative and Preventive Dentistry, Center for Dental Medicine, University of Zürich, Plattenstrasse 11, CH-8032 Zürich, Switzerland. Contributed substantially to discussion, proofread the manuscript.; d Florian J. Wegehaupt Professor and Head Division of Preventive Dentistry and Oral Epidemiology, Clinic of Conservative and Preventive Dentistry, Center for Dental Medicine, University of Zürich, Plattenstrasse 11, CH-8032 Zürich, Switzerland. Research idea, hypothesis, experimental design, contributed substantially to discussion and writing the paper, proofread the manuscript.

**Keywords:** oral hygiene, cleaning performance, toothbrush wear, orthodontic appliances

## Abstract

Purpose: To investigate the impact of manual toothbrush usage duration and associated wear on cleaning performance in a tooth model with fixed orthodontic appliances.

Materials and Methods: Black resin teeth with attached brackets were coated with a white layer of titanium dioxide and subjected to brushing using a brushing machine. Two distinct brushing motions, horizontal and circular, were tested. Following each brushing session, the percentage of cleaned areas (total and adjacent to the bracket) was measured to determine the cleaning performance of the toothbrushes. Usage was simulated using a 3D-printed tooth relief with brackets and wire. Cleaning performance was re-evaluated after simulated 2, 4, 6, 12, 18 and 24 months of usage, and toothbrush wear was quantified respectively.

Results: Cleaning performance of all investigated brushing motions and tooth areas improved during the test period, although statistical significance was only reached for horizontal brushing. Furthermore, horizontal brushing proved more effective regarding total tooth area and the area adjacent to the bracket compared to circular brushing.

Conclusion: This *in-vitro* data shows that toothbrushes may feature sufficient or even improved cleaning performance on teeth with orthodontic appliances even after 24 months. However, direct transferability into the clinical setting is limited, as *in-vivo* toothbrush wear is complex and depends on individual patient habits, and other factors might necessitate an earlier toothbrush change. Nevertheless, this study suggests that cleaning performance and thus oral hygiene in patients with orthodontic appliances may not be critically dependent on usage duration and visual appearance of the toothbrush itself.

Dental plaque is considered the causative agent of major dental diseases, such as caries and periodontal disease.^3,19^ Therefore, the mechanical removal of dental plaque plays an important role in maintaining oral health.^17,21^ Individuals undergoing orthodontic treatment with fixed appliances face increased challenges in maintaining proper oral health,^26,48^ as these appliances create additional surfaces and bacterial niches promoting plaque accumulation.^2,29^ Additionally, plaque removal is significantly impaired by the orthodontic brackets, bands, wires and ligatures, leading to an increased level of cariogenic bacteria in the biofilm.^20,42^ Due to these challenging conditions for adequate oral hygiene, orthodontic patients have a significantly increased risk of developing enamel decalcification^26,48^ and gingival inflammation.^1,12,39^

Various oral hygiene aids are available for mechanical plaque reduction. To date, plaque removal using a toothbrush and fluoridated toothpaste is still the most common and effective method.^11,45,47^ However, the toothbrush undergoes daily usage, resulting in bristle fatigue and irreversibly bent filament tips.^18^ Several methods have been developed to quantify this phenomenon,^8,16,30^ with the wear index and wear rate according to Rawls et al^33^ being commonly used.

It is generally assumed that a toothbrush loses its effectiveness with increasing signs of wear.^18^ However, controversial results have been obtained from *in-vitro* studies and clinical trials that have investigated how toothbrush wear affects plaque removal. While some studies show a decrease in cleaning performance with increased toothbrush wear,^5,8,16,28^ others observed no effect^38,41^ or even an improvement in cleaning performance over time.^6,30,50^

Regarding toothbrush wear in an orthodontic setting, no evidence is available to date. With regard to patients undergoing orthodontic treatment, it is presumed that the toothbrushes experience greater wear due to the higher stress exerted by the fixed appliances on the bristles.^9^ However, the effect of the wear of manual toothbrushes on their cleaning performance when used on teeth undergoing orthodontic treatment has not been investigated so far.

To close this evidence gap, the purpose of this study was to assess how in teeth with fixed orthodontic appliances, the simulated usage of manual toothbrushes influences their cleaning performance.

The null hypothesis was that the duration of use and associated wear of toothbrushes have no influence on their *in-vitro* cleaning performance in a tooth model with fixed orthodontic appliances.

## MATERIALS AND METHODS

### Preparation of Models and Toothbrushes

In order to assess the cleaning performance of the toothbrushes, tooth models with teeth made from black resin (in-house production, University of Zürich, Switzerland) representing the second quadrant were used. The tooth models were made of polyurethane (Siladent; Goslar, Germany) and were cast using a silicone mould based on the morphology of Frasaco plastic teeth (Frasaco; Tettang, Germany). The tooth models included teeth 23 (canine), 24 and 25 (premolars) and 26 to 28 (molars). However, only teeth 24 to 26 were relevant for this experiment.

**Fig 1:**
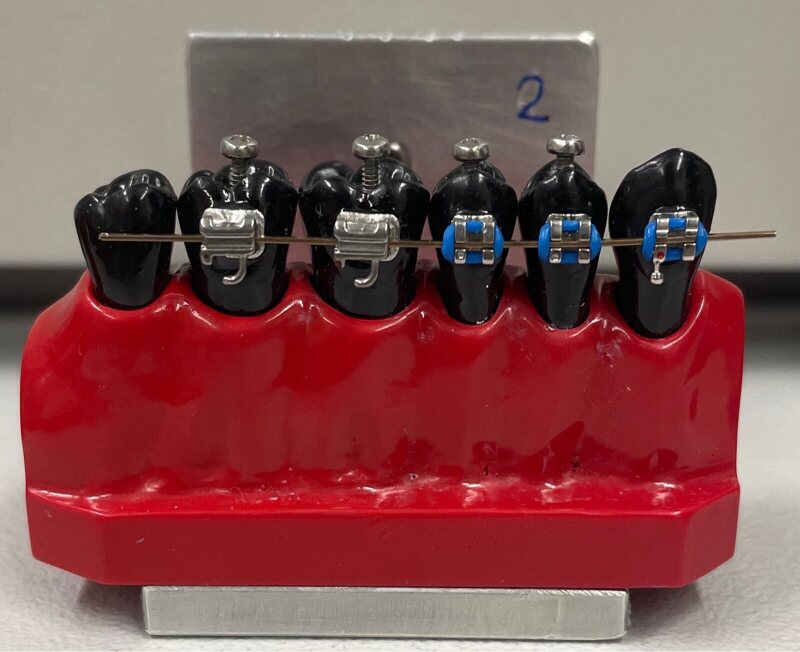
Tooth model with black resin teeth with brackets and inserted wire.

Brackets (Ormco; Glendora, CA, USA) were placed on the buccal surfaces of teeth 23, 24 and 25, while tubes (3M; Saint Paul, MN, USA) were affixed to the buccal surfaces of teeth 26 and 27, using a bracket positioning gauge (Forestadent; Pforzheim, Germany). To attach the brackets and tubes to the teeth, a 2 x 3 mm area was first roughened on the buccal surface of the teeth using a diamond drill (Jota; Rüthi, Switzerland). A light-curing bonding agent (Heliobond, Ivoclar Vivadent; Opfikon, Switzerland) was then applied and cured for 20 s. The bonding surface of the brackets and tubes was conditioned by applying a one-component bonding agent (Monobond Plus, Ivoclar Vivadent; Opfikon, Switzerland), which was left to dry for 1 min. This was followed by applying and curing the light-curing bonding agent Heliobond (Ivoclar Vivadent; Opfikon, Switzerland) for an additional 20 s. Next, the brackets and tubes were bonded to the teeth with a dual-curing composite (Variolink, Ivoclar Vivadent; Opfikon, Switzerland), which was also light-cured for 20 s. Finally, a rectangular 0.016” × 0.022” stainless-steel wire (G&H Orthodontics; Franklin, IN, USA) was inserted into the brackets and tubes and was attached using elastic rubber ligatures (G&H Orthodontics; Franklin, IN, USA) (Fig 1).

The toothbrush Paro M43 (Esro; Kilchberg, Switzerland) was used for this experiment, featuring a flat and parallel bristle field organised in four rows with a total of 43 filament bundles. The rounded ends of the nylon filaments are classified as hardness grade medium. The toothbrush heads of six Paro M43 toothbrushes were separated from their handle and then glued to clampable aluminium rods (in-house production, University of Zürich, Switzerland) to allow for use in the brushing machines ZMB2 and ZMB8 (both in-house production, University of Zürich, Switzerland), using an instant adhesive (Loctite 480, Henkel; Düsseldorf, Germany).

### Test Procedure

**Fig 2:**
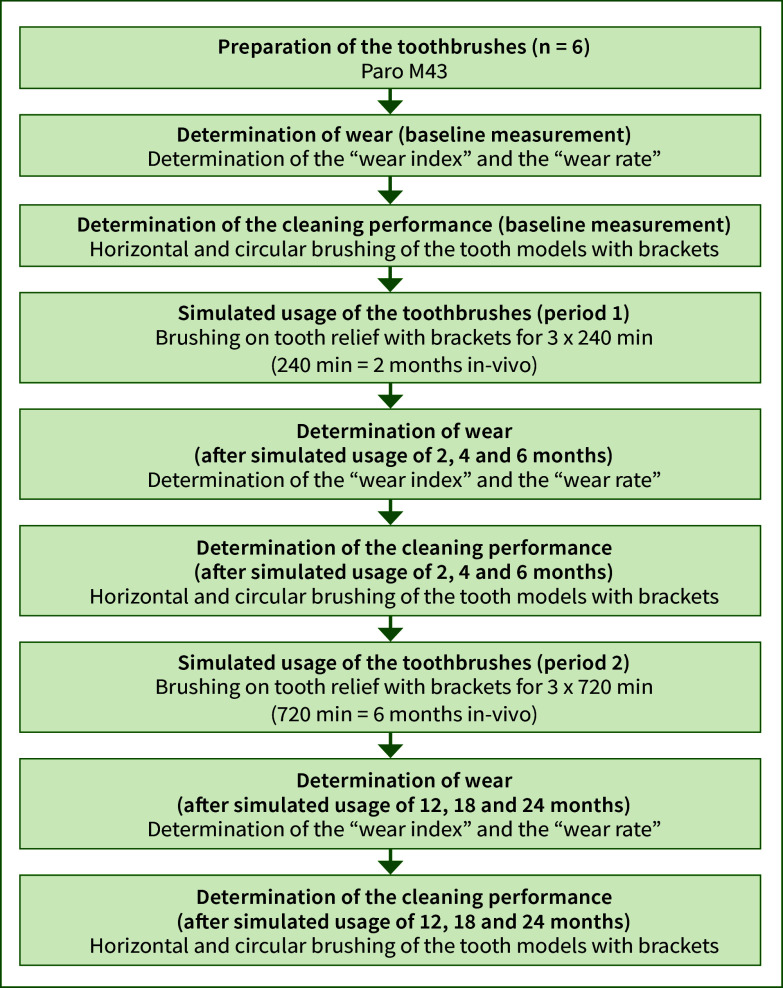
Schematic representation of the test procedure.

Wear index and wear rate were initially determined for six new toothbrushes (n = 6). Then, their cleaning performance was assessed by employing three identical tooth models, each of which was assigned two toothbrushes (toothbrush 1 + 2 on tooth model A, toothbrush 3 + 4 on tooth model B and 5 + 6 on tooth model C). These initial evaluations, conducted at time point 0, served as the baseline measurements for the subsequent analysis. Afterwards, toothbrush usage was simulated by brushing over a 3D-printed tooth relief with brackets and a wire, simulating a total period of 24 months. The simulated usage spanned two distinct periods: the first lasting 6 months with intervals of 2 months and the second extending over 18 months with intervals of 6 months. At intervals of 2, 4, 6, 12, 18 and 24 months of simulated usage, new measurements for the wear index, wear rate and cleaning performance were conducted. The specific test procedure is illustrated in Figure 2. The determination of the wear index and wear rate, the evaluation of cleaning performance, as well as the simulation of toothbrush usage are further detailed in subsequent sections.

### Determination of the Cleaning Performance

To determine the cleaning performance of the toothbrushes, the tooth models were coated with a white layer of titanium dioxide before each brushing procedure. For this purpose, a 26% ethanol solution (Reuss-Chemie; Tägerig, Switzerland) and titanium dioxide (Merck; Darmstadt, Germany) were mixed in a mass ratio of 2:1. Teeth 24 to 26 were coated by hand with the titanium dioxide using a brush, similarly to previous studies.^4,43,44,50^ The teeth coated with the titanium dioxide were then left to dry for 30 min.

For the brushing procedure, the toothbrush heads attached to the aluminium rods were clamped in the ZMB2 brushing machine so that the centre of the toothbrush was aligned with the centre of the corresponding tooth model. A contact pressure of 2.5 N was set using a spring balance (Pesola; Schindellegi, Switzerland). The cleaning performance of the toothbrushes was tested using two different brushing motions: horizontal and circular brushing. The teeth were brushed at a frequency of 60 cycles/min back and forth for horizontal brushing, while for circular brushing a circular motion of 60 cycles/min was added to the 60 cycles/min for horizontal brushing. Toothpaste was deliberately omitted from the brushing procedure, to prevent dissolution of the titanium dioxide in water. Although titanium dioxide does not fully represent the mechanical properties of dental biofilm, it still allows the determination of the contact area between the tooth surface and the toothbrush. Consequently, black areas following the respective brushing procedure were interpreted as areas touched by the bristles of the toothbrushes and thus considered cleaned.^13^

**Fig 3:**
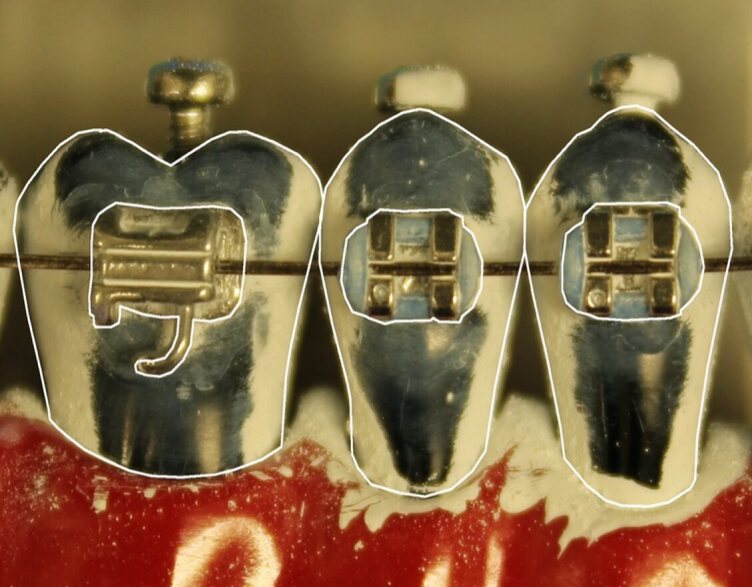

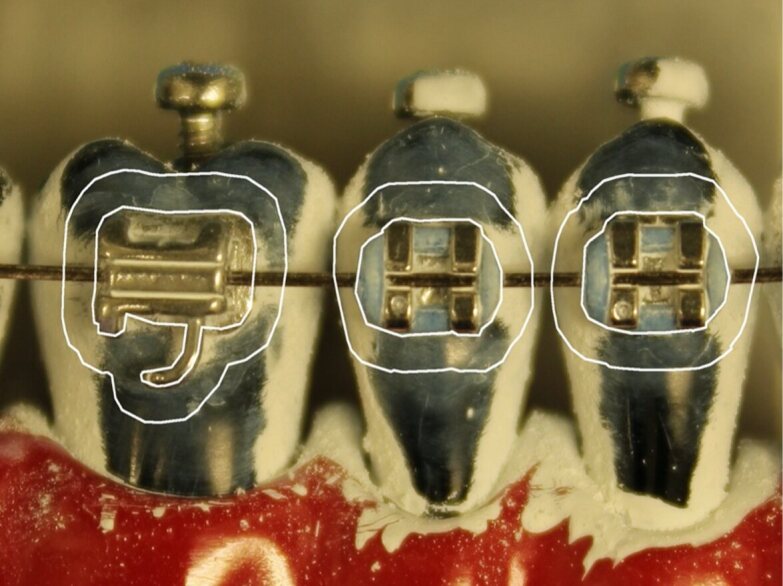
Masks for the determination of the cleaned areas are superimposed on the respective pictures. Left: mask for the entire (total) tooth surface; right: mask for the 1 mm-wide ring surrounding the bracket.

Following each brushing of a model, the extent of the titanium dioxide layer removed from the tooth surfaces (teeth 24 to 26) was determined, indicating the cleaning performance of the toothbrushes. For this purpose, the tooth models were digitally photographed after each brushing session, and the images were analysed using the Fiji program (Fiji Team, https://fiji.sc/).^37^ To quantify the area cleaned by the toothbrushes on the model teeth, masks were superimposed on the teeth (24, 25 and 26) in the corresponding images. For each tooth in the three models, there were two distinct masks: one for the entire tooth surface (later referred to as total area) and another for a 1 mm-wide ring surrounding the bracket (later referred to as ring area) (Fig 3). The use of the Fiji program allowed to determine the percentage of the total and ring areas that had been cleaned.

After each brushing session and the subsequent digital image capture, the teeth were cleaned with water and soap. The tooth models were then repainted in preparation for the next brushing process.

### Simulation of Usage of the Toothbrushes

To simulate the usage experienced by toothbrushes during daily use, the toothbrushes (n = 6) were worn down by brushing over a 3D-printed tooth relief (in-house production, University of Zürich, Switzerland) with brackets and wire.

**Fig 4:**
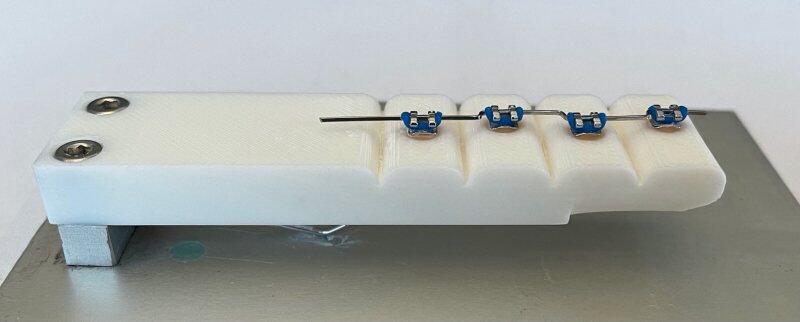
3D-printed tooth relief with brackets and wire used for the simulated usage of the toothbrushes.

The tooth reliefs, designed to mimic four adjacent teeth (waves), were made from a polylactide (PLA) plastic using a 3D printer (Bambu Lab X1 Carbon, Bambu Lab; Austin, TX, USA). A bracket was placed on each of these waves at a distance of 1 mm, alternately to the right or left of the centreline. This staggered arrangement was chosen to simulate wear along the whole width of the toothbrush head. The brackets were attached to the waves of the tooth relief using the same procedure that was used to bond the brackets to the tooth models. The same stainless-steel wire used for the tooth models was then inserted into the brackets with elastic rubber ligatures (Fig 4).

After determining the cleaning performance of the toothbrushes on the tooth models at a given measurement time point, the toothbrushes were each clamped in the brushing machine ZMB8 to simulate wear. The toothbrushes were centrally aligned on the tooth reliefs. A contact pressure of 2.5 N was checked using a spring balance. Subsequently, containers, each containing 72 g of toothpaste slurry, were placed in the brushing machine, such that the toothbrushes and tooth reliefs were surrounded by slurry. The toothpaste slurry was freshly prepared just before the wear process in a ratio of 2:1 from artificial saliva, according to Klimek et al^14^ and Elmex toothpaste (GABA; Therwil, Switzerland). The brushing machine brushed over the tooth reliefs with the brackets at a frequency of 60 cycles/min with reciprocating back-and-forth movements. During the initial simulated usage period, which spanned 6 months with intervals of 2 months, each usage simulation session lasted for a duration of 240 min. Subsequently, in the second period, a usage simulation covering 18 months was conducted, with intervals of 6 months (each usage simulation session lasting for a duration of 720 min).

At the end of each wear process, the toothbrushes were cleaned with water and dried. The state of wear and cleaning performance of the toothbrush were then assessed.

### Determination of Wear Index and Wear Rate

In order to quantify the bristle splaying and the state of wear of the toothbrushes, the wear index, according to Rawls et al^33^ was recorded at the beginning of the entire experiment (new brush = time point 0) and after the simulation of 2, 4, 6, 12, 18 and 24 months (time points 1–6) of in-vivo toothbrushing. In addition, the overall state of deterioration of the toothbrushes was assessed using the wear rate at each of these time points.^33^ All measurements for the wear index and the evaluation of the wear rate of the individual toothbrushes were carried out by one observer.

To determine the wear index, the aluminium rods with the attached toothbrush heads were fixed in a custom-made cast made of impression material (President Original Light Body, Coltene; Altstätten, Switzerland) at each of the measurement time points. The toothbrush heads were photographed from both the lower end of the toothbrush head and the side using a Canon EOS 200D camera (Canon; Tokyo, Japan). The brush silhouettes were then evaluated using the Fiji program. Based on the silhouette images, the width of the bristle field at both the attached and free ends was measured for each toothbrush at each time point, for the long (Wf^L^ and Wa^L^) and short sides (Wf^S^ and Wa^S^), as well as the length of the bristles (Lo). The wear index (WI) was then calculated according to the formula proposed by Rawls et al.^33^


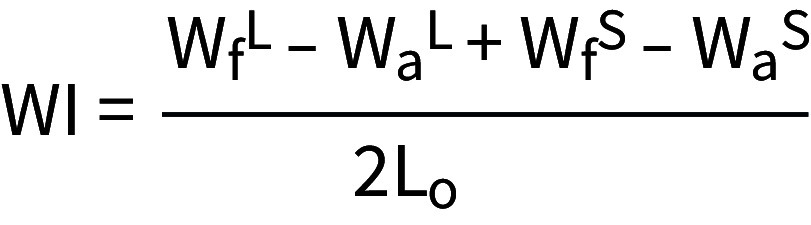


To determine the wear rate of the toothbrushes according to Rawls et al,^33^ the subjective rating scale consisting of four levels and ranging from 0 to 3 was applied. In case of ambiguity, the higher value was chosen.

### Statistical Analysis

Statistical analysis was performed using the statistical software R,^31^ including the package tidyverse.^46^

Descriptive statistics (median/IQR) for the cleaned areas were calculated for the respective time points of measurement (duration of simulated toothbrush usage). The total and ring areas were analysed independently, each for both horizontal and circular brushing. To evaluate if there is a significant change in the cleaned area after simulated usage of the toothbrush, the respective maximum values for every combination of toothbrushing motion and tooth area (total and ring) were statistically compared with the corresponding baseline values using the Wilcoxon signed rank exact test. The level of significance was set at p < 0.05.

In order to quantify toothbrush wear, descriptive statistics (median/IQR) for WI and wear rate were calculated.

## RESULTS

The values determined for the cleaned surface per tooth area and brushing motion are provided in Table 1. An overview of the WI and wear rate for the respective months of simulated usage is given in Table 2.

**Table 1:** Median/IQR values of the cleaned surface [%] after different durations of simulated usage of the toothbrushes (n = 6) for the different investigated areas and brushing motions.

	Duration of simulated usage [months]
0	2	4	6	12	18	24
Cleaned area (median/IQR) [%]	Horizontal brushing	Total	59.30/2.80	69.20/3.18	71.65/2.85	72.25/6.25*s	67.90/3.23	69.95/2.90	70.05/1.33
Ring	59.27/3.30	63.75/1.39	64.18/1.48	65.56/1.16	65.39/4.17	66.78/5.23*s	66.39/2.57
Circular brushing	Total	61.40/9.28	65.10/2.95	64.15/1.78	67.55/2.50*	66.10/1.63	67.00/3.20	65.70/3.45
Ring	50.37/8.62	55.67/3.56	55.14/4.43	59.09/2.60*	58.96/2.75	58.51/1.41	58.21/4.76
For each brushing motion and tooth area the maximum value of the cleaned area was marked with*. If there was a statistically significant difference (p < 0.05) between the respective maximum and the corresponding initial value (0 months), then these values were marked with s.

**Table 2:** Median/IQR values for wear index and wear rate after the respective months of simulated usage.

	Duration of simulated usage [months]
0	2	4	6	12	18	24
Wear index (median/IQR)	0.055/0.018	0.100/0.008	0.100/0.065	0.110/0.028	0.115/0.018	0.125/0.025	0.125/0.018
Wear rate (median/IQR)	0.0/0.00	2.0/0.00	2.0/0.00	2.0/0.75	2.0/0.75	2.5/1.00	3.0/0.00


### Cleaning Performance

For horizontal brushing, the maximum of the total cleaned area (median/IQR) was observed after 6 months of simulated usage of the brushes (72.25%/6.25%), which was significantly higher (p < 0.05) compared with the respective baseline value (59.30%/2.80%). For the cleaned ring area, a maximum was observed after 18 months of simulated usage (66.78%/5.23%), which was again significantly higher (p < 0.05) compared with the respective baseline value (59.27%/3.30%).

However, for circular brushing, regarding the total cleaned area, the maximum value observed after 6 months (67.55%/ 2.50%) was not significantly higher (p > 0.05) compared with the respective baseline value (61.40%/9.28%). For the cleaned ring area the maximum value was also observed after 6 months (59.09%/2.60%), however this was again not significantly higher (p > 0.05) compared with the respective baseline value (50.37%/8.62%).

Furthermore, regarding descriptive statistics, it was observed that for the same brushing motion, the total area was always better cleaned than the respective ring area. Additionally, after simulated usage, when brushing horizontally, the total or ring areas were better cleaned than the respective areas when performing circular brushing.

### Wear Index and Wear Rate of Toothbrushes

The highest increase in WI was observed during the first 2 months of simulated usage (between baseline [0 months] and 2 months). During further usage, the WI increased steadily but slowly.

Similarly, concerning the wear rate, an initial increase was observed between baseline (0 months) and 2 months. However, in the following, it only increased again after 18 and 24 months of simulated usage.

## DISCUSSION

The purpose of this *in-vitro* study was to assess how in teeth with fixed orthodontic appliances, the duration of use and associated wear of manual toothbrushes influence their cleaning performance. This study presents the first data on toothbrush wear regarding teeth with orthodontic brackets.

For this *in-vitro* analysis on tooth models with fixed orthodontic appliances, well-established methods^13^ were used, to simulate the usage of toothbrushes and assess their cleaning performance. The cleaning performance of two different cleaning methods (circular or horizontal brushing) was compared and individually analysed for the total tooth area as well as for a 1 mm ring area surrounding the bracket. In order to quantify the state of wear of the toothbrushes, the WI and wear rate, according to Rawls et al^33^ were recorded for the respective time points.

Due to the *in-vitro* approach, the present study offers valuable insights into the impact of toothbrush wear on cleaning performance in teeth with fixed orthodontic appliances. The experimental conditions allow for a certain level of standardisation and results are likely to be reproducible. However, the transferability into the clinical setting is limited. A significant limitation is the artificially induced wear of the toothbrush bristles, which only approximates the characteristics of naturally worn toothbrushes. It is likely that other factors such as oral microorganisms, food particles, toothpaste abrasiveness and the natural ageing of the bristles over time contribute to the *in-vivo* wear of toothbrushes. Furthermore, the wear of the bristles depends on individual factors, including toothbrushing technique, contact pressure, brushing duration and individual habits, in particular chewing on the toothbrush.^15,16,22^ Regarding orthodontic patients, the geometry of brackets and wires could further influence both toothbrushing performance and wear. As of many commercially available systems for fixed orthodontic appliances only one was considered in this experiment, different systems may lead to other behaviour regarding cleaning performance and toothbrush wear.

There is controversial evidence regarding the efficacy of different toothbrushing methods,^32^ especially in teeth with fixed orthodontic appliances.^23,25^ The present study investigated horizontal and circular brushing, as these techniques are commonly used.^7,13,24^

Based on clinical studies a contact pressure of 2.5 N was applied on the toothbrush.^7^ The calculations for simulated usage duration were based on the assumption that the majority of the population brushes their teeth at least twice a day for 2 min each time.^40^ This results in a total duration of 120 min *in-vivo *toothbrushing in one month. The corresponding usage duration at different measurement time points (after 2, 4, 6, 12, 18 and 24 months) resulted from multiplying the monthly usage time of a toothbrush (120 min) by the number of simulated months.

The present study utilised Paro M43 toothbrushes which are listed as a reference toothbrush by the American Dental Association (ADA). Paro M43 toothbrushes have frequently been applied in other studies, predominantly focused on cleaning or abrasion tests.^34,36,49^ However, there are several other studies that have used different types of toothbrushes.^38,41^ It is evident that the results of this *in-vitro* study cannot adequately represent the diversity of toothbrushes available on the market, as the study’s findings are specific to toothbrushes with a plane brush-head design, such as Paro M43.

Another restriction of this study was the titanium dioxide coating, which was used as a plaque substitute. There is no standardised procedure for creating artificial plaque. However, the titanium dioxide coating used in this study has previously been described by Imfeld et al^13^ and several other studies.^35,36,43^ As the titanium dioxide coating is only removed from the areas effectively touched by the filaments and does not flake off uniformly, the surfaces freed from the coating were considered to have come into contact with the brush and thus hypothetically cleaned. This plaque substitute does not entirely fulfil the physical characteristics of dental plaque, such as viscosity, water insolubility, and high abrasion resistance. Toothpaste was intentionally omitted from the brushing procedure, as the titanium dioxide would have dissolved in water. Despite this limitation, the methodology adopted in this study proves effective in representing the contact areas between the toothbrush and the tooth model, providing insights into the potentially cleaned areas.

It is commonly assumed that a toothbrush loses its effectiveness as signs of wear increase.^18^ Concerning patients undergoing orthodontic treatment, the assumption is that the bristles experience greater wear due to increased stress from the fixed orthodontic appliances.^9^ For horizontal brushing, the present study observed a statistically significant increase in the *in-vitro* cleaning performance for both the total and the ring areas, as the toothbrush experienced wear compared to the new toothbrush. A similar increase was observed for both areas after circular brushing, although statistical significance was not reached. These findings align with a prior study employing a similar setup, investigating teeth without orthodontic appliances, where an increase in cleaning performance was observed as the toothbrush underwent wear.^50^

In detail, a maximum in cleaning performance was reached at 6 months (time point 3) for the total area with horizontal brushing and the total and ring area with circular brushing. The peak for the ring area with horizontal brushing was reached at 18 months (time point 5). Subsequently, as wear progressed, cleaning performance decreased to a plateau until 24 months (time point 6), although it remained above the initial cleaning performance (time point 0). A possible explanation for this observed phenomenon, previously described by Zoller et al,^50^ argues that as the bristles undergo wear and interact with the toothpaste slurry, they become more flexible, allowing for better adaptation to the tooth surface contours and improved cleaning of outer areas, including those around the bracket. However, after reaching a peak in cleaning performance at 6 or 18 months, the subsequent decrease and plateau may be attributed to the further fraying of the bristles with increasing wear, so that the applied contact pressure of 2.5 N might no longer be sufficient to bring the bristles into sufficient contact with the tooth surface for effective cleaning.

**Fig 5:**
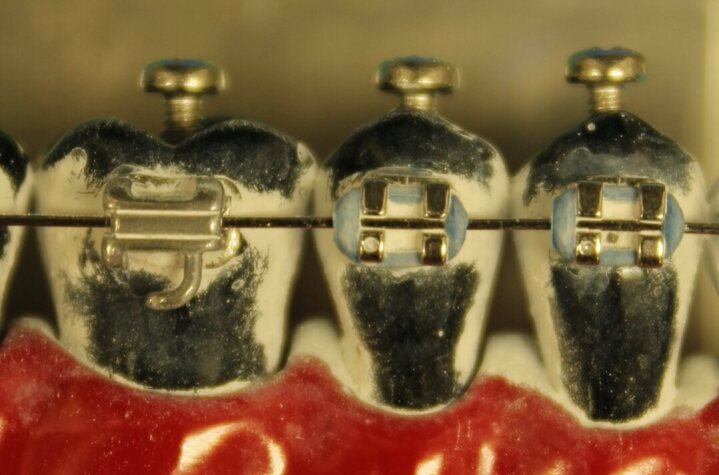

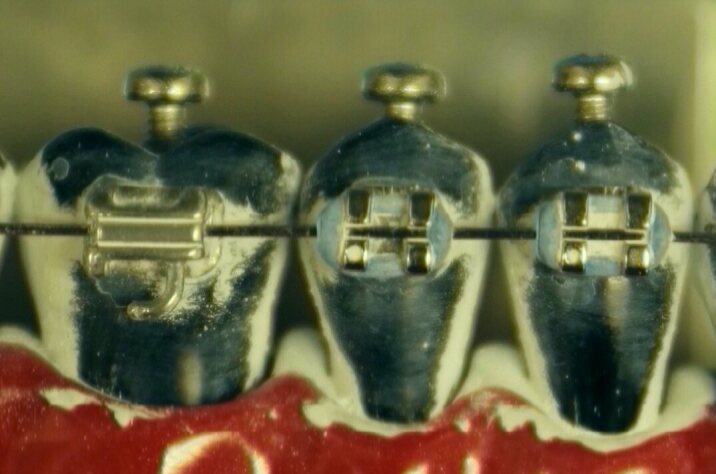
Representative presentation of the cleaned areas after circular (left) and horizontal (right) brushing.

Furthermore, for horizontal brushing, both the total and the ring areas were cleaned more effectively compared to circular brushing, after simulated usage. A hypothesis for these results is that during horizontal brushing the bristles potentially miss the area straight below the wire but may effectively clean the area beside it. In contrast, while brushing circularly the bristles consistently leap over the wire, leading to a skipped area around the wire where the bristles do not touch the tooth. Therefore, as depicted in Figure 5, the circular brushing creates a larger region around the wire that does not get cleaned by the bristles in comparison to the horizontal brushing.

Additionally, it was observed that within the same brushing motion, the total area consistently showed better cleaning than the respective ring area. It is presumed that the area directly adjacent to the bracket is challenging for toothbrush tufts to reach, as the brackets create obstacles for the bristles. This results in the smaller ring area around the brackets being proportionally less cleaned than the total area. This is in line with clinical findings, which indicate that fixed orthodontic appliances create retention niches, increasing the risk of caries and enamel demineralisation around the brackets.^2,10,27^

**Fig 6:**
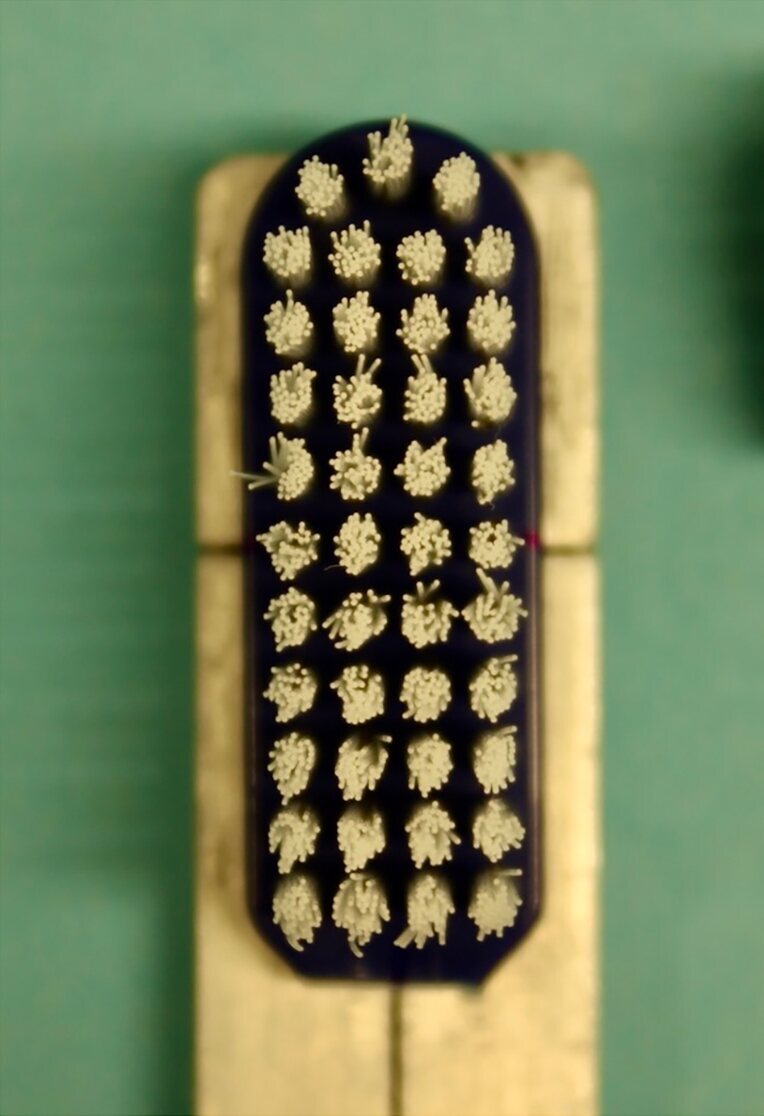

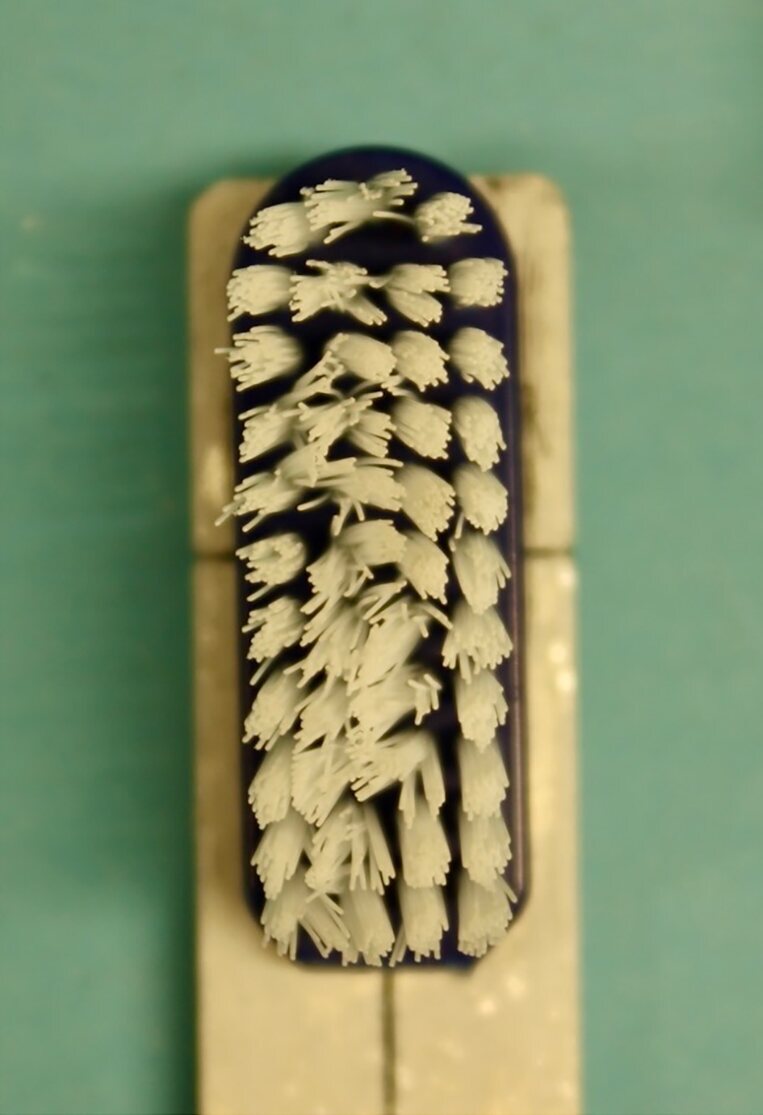
Left: new toothbrush; right: toothbrush appearance after 24 months of simulated usage.

In general, no evidence regarding toothbrush wear in an orthodontic setting is available to date. Concerning teeth without orthodontic appliances, evidence on the cleaning performance of toothbrushes with increasing wear is inconclusive, with certain studies showing an improvement in cleaning performance,^6,30^ no effect of toothbrush wear^38,41^ or even a decrease in cleaning performance.^5,8,16,28^ Nevertheless, the comparability of the studies is limited, as they comprise *in-vitro* and *in-vivo* studies. Furthermore, the variations in the results among the mentioned studies may also be attributed to factors such as the type of toothbrush, the brushing duration, the test arrangements and the method applied to determine and calculate toothbrush wear. In this context, it is also noticeable that in certain studies^28,38,41^ the toothbrushes showed more visible signs of wear after a certain usage period compared to the present study (Fig 6).

## CONCLUSION

In conclusion, this *in-vitro* study revealed an improvement in cleaning performance on teeth with fixed orthodontic appliances, as the toothbrushes underwent wear compared to new ones. To date, there are no publications on toothbrush wear in orthodontics. The results of this study challenge general guidelines on changing toothbrushes every 3 months as recommended by the ADA. Although this *in-vitro* data suggests that toothbrushes may feature a sufficient or even improved cleaning performance even after 24 months, it must not be concluded that no toothbrush replacement is necessary for this period, as *in-vivo* toothbrush wear is critically dependent on individual patient habits such as toothbrushing technique, and as other factors may necessitate an earlier toothbrush exchange, mainly microbial colonization and the risk of gingival injury with increasing wear of the bristles.^15^ Nevertheless, this study suggests that cleaning performance and thus oral hygiene in patients with orthodontic appliances may not be critically dependent on the usage duration and visual appearance of the toothbrush itself. The observation that, in the present study, toothbrushes with similar wear levels exhibited better cleaning results for horizontal brushing than circular brushing implies that the cleaning technique might play a pivotal role in overall cleaning performance. While the study indicates a positive impact of usage duration and wear on toothbrush performance, other factors, such as toothbrushing technique and individual habits, might exert a more significant influence on *in-vivo* cleaning performance and oral hygiene than the duration of toothbrush wear or its appearance.

### Acknowledgements

The study was conducted as the doctoral thesis of med. dent. Florance A. Lasance and performed at the Center for Dental Medicine of the University of Zürich, Switzerland, under the supervision of Prof. Dr. F. J. Wegehaupt and Prof. Dr. Dr. h.c. T. Attin.
